# Discrepancy Between Vaccination Willingness and Actual SARS-CoV-2 Vaccination Status in People with Multiple Sclerosis: A Longitudinal Study

**DOI:** 10.3390/jcm14113689

**Published:** 2025-05-24

**Authors:** Felicita Heidler, Michael Hecker, Niklas Frahm, Julia Baldt, Silvan Elias Langhorst, Pegah Mashhadiakbar, Barbara Streckenbach, Katja Burian, Jörg Richter, Uwe Klaus Zettl

**Affiliations:** 1 Department of Neurology, Ecumenic Hainich Hospital gGmbH, 99974 Mühlhausen, Germany; jrichterj@web.de; 2 Department of Neurology, Jena University Hospital, 07747 Jena, Germany; 3 Division of Neuroimmunology, Department of Neurology, Rostock University Medical Center, 18147 Rostock, Germany; michael.hecker@med.uni-rostock.de (M.H.); niklas.frahm@med.uni-rostock.de (N.F.); julia.baldt@uni-rostock.de (J.B.); pegah.mashhadiakbar@outlook.de (P.M.); barbara.streckenbach@uni-rostock.de (B.S.); katja.burian@uni-rostock.de (K.B.); uwe.zettl@med.uni-rostock.de (U.K.Z.); 4 Faculty of Health Sciences, University of Hull, Hull HU6 7RX, UK

**Keywords:** multiple sclerosis, SARS-CoV-2 pandemic, vaccination willingness, vaccination status

## Abstract

**Background/Objectives:** Infection with severe acute respiratory syndrome coronavirus type 2 (SARS-CoV-2) poses a significant health risk, especially for individuals with chronic medical conditions. Multiple sclerosis (MS) is the most prevalent chronic, immune-mediated neurological disorder, and vaccinations are essential to its management. This study aimed to compare the reported willingness to be vaccinated against SARS-CoV-2 with the actual vaccination status among people with MS (pwMS) and identify factors explaining the discrepancy. **Methods**: In a longitudinal, two-center study, we analyzed 149 patients aged 18 or older with a diagnosis of clinically isolated syndrome or MS. The participants completed three surveys: a baseline survey (from June 2019 to June 2020), a pre-vaccine follow-up (from May to July 2020), and a post-vaccine follow-up (from October 2021 to January 2022). The data included sociodemographic, clinical, and psychological information. **Results**: Among the 149 participants, 122 (81.9%) received a SARS-CoV-2 vaccination, while 27 (18.1%) did not. The pwMS who were unwilling to become vaccinated and remained unvaccinated were less likely to live with a partner, had higher smoking rates, took more medications, had a higher number of previously discontinued disease-modifying therapies, and found pandemic policies inappropriate. No significant associations were found between vaccination willingness/status and factors like age, sex, depression, or anxiety. **Conclusions**: This study highlights the gap between vaccination willingness and actual status in pwMS, revealing factors associated with vaccine hesitancy. The findings of this study offer insights into addressing vaccine uptake.

## 1. Introduction

The global pandemic of the severe acute respiratory syndrome type 2 (SARS-CoV-2) virus has significantly impacted countries worldwide, making it the most destructive pandemic of the 21st century. On 11 March 2020, the World Health Organization (WHO) declared the SARS-CoV-2 outbreak to be a global health emergency. Although the WHO ended the international health emergency on 5 May 2023, it warned that the virus remains a threat with the potential for new variants. The pandemic has resulted in 7.1 million confirmed deaths, and 13.64 billion people having received a vaccine dose, with 67% of the global population receiving the full initial vaccine course [1].

The outbreak of SARS-CoV-2 infections presented a significant challenge, as patients required hospitalization for acute respiratory distress syndrome and multiple organ failure. The fatality rate was contingent upon the presence of additional risk factors. Patients with autoimmune diseases, such as multiple sclerosis (MS), were particularly vulnerable [2]. MS is the most common chronic inflammatory autoimmune disease of the central nervous system (CNS) that affects the brain and spinal cord in young adults [3,4,5,6]. The disease causes a range of symptoms including muscle weakness, loss of coordination, vision problems, and cognitive impairment. People diagnosed with MS (pwMS) exhibit an increased vulnerability to infections during the course of the disease [2], a susceptibility that can be further compounded by the administration of disease-modifying therapies (DMTs) [7,8,9].

International efforts have been made to clarify the risk of severe SARS-CoV-2 infection in pwMS. In a study by Louapre et al., exposure to DMTs and the level of immunosuppression were not factors that independently changed the risk of developing a severe form of SARS-CoV [10] (odds ratios (Ors): 0.07–0.37) [11], yet this risk may be made more severe by other factors, such as the use of B-cell depleting agents [11,12,13]. On the other hand, patients with progressive MS (OR: 3.74), an older age (OR per 10 years: 1.45), and other comorbidities (such as diabetes, obesity (both OR: 1.87), and cardiovascular disease (OR: 1.96)) were found to be at an even more increased risk of severe infection [10]. The guidelines set by the National MS society during the SARS-CoV-2 pandemic recommended that pwMS should receive a vaccine against SARS-CoV-2 as soon as it became available [14]. For these reasons, vaccinations against infectious diseases are of great importance for pwMS [8,15,16].

The first studies that compared the willingness of pwMS to become vaccinated against SARS-CoV-2 with the actual vaccination rates were published at a time when the vaccine was theoretically available but not yet within practical reach for all study participants. Consequently, there was an initial discrepancy between the reported readiness for vaccination and the actual vaccination rates, and this discrepancy reached up to 42.7% in a study by Huang et al. [17]. This US research group found a vaccination readiness rate of 94.4% and a vaccination rate of 51.7%. Similar discrepancies were reported by Uhr and Mateen [18] (76.6% vaccination willingness vs. 54.4% vaccination reality) as well as Wu et al. [19] (66.0% vaccination willingness vs. 48.4% vaccination reality). By mid-2021, the SARS-CoV-2 vaccination rate was 82.9% in an Australian MS cohort, as reported by Day et al. [20], and 61% in an Iranian study by Nabavi et al. [21]. The considerable discrepancy between the stated willingness to be vaccinated against SARS-CoV-2 and the actual receipt of a vaccination can also be found in the general population. Despite the availability of vaccines, many countries have reported slow uptake and significant vaccine hesitancy, even among vulnerable populations [22,23]. Vaccine hesitancy, defined as delay in accepting or refusing a vaccine despite its availability, is a long-standing issue that has been reported in various studies [24]. The WHO has identified vaccine hesitancy as one of the top 10 threats to global health in 2019 [25]. During the pandemic, SARS-CoV-2 vaccine hesitancy rates reached up to 50% of the general population, with significant regional variation [18,22,23,26]. Factors that are commonly associated with vaccine hesitancy include a younger age, lower education and income levels, previous anti-vaccine beliefs, and low trust in the government [18,22,23]. According to the survey of Dror et al. [27], the most significant positive predictor for acceptance of a SARS-CoV-2 vaccine in the general population was current influenza vaccination [17,18,19,28,29,30]. Reasons for vaccine hesitancy specifically in the MS population have not been clearly delineated and further research is required.

The present study had two main objectives. The first was to compare the initial willingness to become vaccinated against SARS-CoV-2 with the later vaccination status in pwMS. The second was to examine associations between vaccination willingness/status and various patient-specific factors, including sociodemographic and clinical data, personality traits, comorbidities—particularly depression and anxiety symptoms—and polypharmacy. By identifying factors that influence vaccination willingness/status, our study aims to contribute to the development of approaches that may enhance vaccine acceptance among pwMS.

## 2. Materials and Methods

### 2.1. Study Population and Inclusion Criteria

This study was performed at two MS centers in Germany (Department of Neurology at the Rostock University Medical Center and Department of Neurology of the Ecumenical Hainich Hospital in Mühlhausen) and was conducted in accordance with the Declaration of Helsinki. The ethics committees of Rostock’s University Medical Center (permit number A 2019-0048) and the Physician’s Chamber of Thuringia approved the study. The consent form for participation was distributed to all participants and signed.

Patients were included if they met the following criteria: age of at least 18 years and diagnosis of a clinically isolated syndrome (CIS) or MS according to the revised McDonald criteria from 2017 [5]. Pseudonymization was ensured by labeling questionnaires and interview sheets with numbers instead of identifying information of the participants.

### 2.2. Data Acquisition

The data were acquired during three subsequent periods:

- From June 2019 to June 2020 (baseline survey) [31];
- From May to July 2020 (first follow-up: approximately 3 months after the SARS-CoV-2 outbreak was declared a global pandemic, before vaccines against SARS-CoV-2 were available) [32];
- From October 2021 to January 2022 (second follow-up: approximately 1 year after the market entry of the first SARS-CoV-2 vaccines in Germany) [33].

The baseline survey was conducted at outpatient clinics during patients’ medical appointments (outpatients) and at neurological wards (inpatients). Data pertaining to the patients’ sociodemographic characteristics, clinical and neurological status, medication regimen, and vaccination history were collected through a structured interview, a medical history review, and a review of the patient’s medical records. Furthermore, psychological data were gathered using the NEO Five-Factor Inventory (NEO-FFI), the Temperament and Character Inventory-Revised (TCI-R), and the Hospital Anxiety and Depression Scale (HADS), as detailed in the following subsection. Follow-up surveys were conducted via questionnaires in outpatient clinics and through telephone interviews.

#### 2.2.1. Data Gathered Before the SARS-CoV-2 Outbreak (Baseline)

The sociodemographic dataset includes information on age, sex, duration of school education, level of post-school education (no training, skilled worker, technical college, university), employment status (full-time work, part-time work, unemployed, old-age pension, disability pension), place of residence (rural community, provincial town, medium-sized town, city), partnership status (single, any partnership), number of children as well as siblings, and smoking habits (current smoking status, ever smoking status, age when smoking was started).

The clinical-neurological dataset comprises the number and type of comorbidities [34] (classified into cancer, cardiovascular, chronic inflammatory, dermatological, ear-nose-throat, endocrinological, gastrointestinal, hematological, metabolic, neurological, ophthalmological, orthopedic, pain, psychiatric, pulmonary, urological, other), the degree of neurological disability (measured via the Expanded Disability Status Scale (EDSS) [35]), the MS course type (CIS, relapsing-remitting MS (RRMS), primary progressive MS (PPMS), secondary progressive MS (SPMS)) [36], and the disease duration (i.e., the time from MS diagnosis to the baseline survey).

In the medication profile, the drugs that were actually used were classified according to three criteria: therapy duration (long-term or on-demand), prescription status (prescription or over-the-counter), and treatment aim (DMT, symptomatic therapy, treatment of comorbidities and further conditions). Furthermore, the total number of drugs was recorded, and patients were classified as either those with polypharmacy (concomitant use of ≥5 drugs) or those without polypharmacy (≤4 drugs) [37]. Information regarding the number of previous DMT switches (with associated reasons for switching) and the frequency of missing medication intake (on a weekly and monthly basis) was obtained.

The dataset included the following variables pertaining to vaccination: the last time the pwMS dealt with the issue of vaccination (last week, last month, last six months, last year, more than a year ago), the person who is most frequently checking their vaccination status (nobody, patient himself, family doctor/company doctor, neurologist, others), whether they received general vaccination advice from a doctor in the past year (yes, no), whether they received advice from a doctor about travel vaccinations (yes, no), whether the patient wants vaccination advice (no, yes (by family doctor, neurologist, or anyone)), the patient’s knowledge about the existence of governmental recommendations for vaccination (yes, no), the patient’s willingness to receive governmentally recommended vaccinations (yes, no), reasons for unwillingness to receive these vaccinations (general refusal of vaccinations, belief that vaccinations are generally ineffective, due to the patient´s medication, concerns about side effects of vaccinations, concerns about vaccine-induced exacerbation of MS), the occurrence of health problems after any vaccination in the past (none, minor vaccination reactions, vaccination complications), the possession of a vaccination card (yes, no), and the patient’s vaccination status with regard to diphtheria, influenza, measles, mumps, pertussis, poliomyelitis, rubella, and tetanus.

The NEO-FFI (German version) was the initial instrument employed in our study to assess personality traits in pwMS. The inventory comprises 60 items which are rated on a 5-point Likert scale ranging from “strongly disagree” to “strongly agree” [38,39]. The five personality dimensions assessed were neuroticism, extraversion, openness, conscientiousness, and agreeableness. In their examination of the applicability of the NEO-FFI in pwMS, Schwartz et al. concluded that the instrument was suitable, reporting good internal consistency, factorial validity, and alignment between self-reports and observer ratings [40].

In the present study, the TCI-R was also utilized for the assessment of personality traits. The German version of the TCI-R employs a true/false response format [41,42,43]. The four temperament dimensions (novelty seeking, harm avoidance, reward dependence, persistence) are typically considered to be relatively stable over time and are thought to reflect automatic emotional responses. Character dimensions (self-directedness, cooperativeness, self-transcendence) are indicative of individual differences in values and norms. The factorial, convergent, and discriminant validity of the TCI-R was confirmed in a study by Farmer et al. [44].

The HADS is a reliable psychological screening tool with strong psychometric properties for assessing anxiety (HADS-A) and depression symptoms (HADS-D) in pwMS [45,46]. It comprises 14 items—7 for anxiety and 7 for depression—and employs a four-point self-report response scale (0–3) [47,48]. HADS scores were classified into three categories: normal (0–7), borderline (8–10), and abnormal (11–21) [49,50]. A cut-off of ≥8 for anxiety and ≥11 for depression symptoms effectively distinguishes between having and not having any psychopathological disturbance in pwMS (HADS-A: 82% sensitivity, 68% specificity; HADS-D: 31% sensitivity, 95% specificity) [48].

#### 2.2.2. Data Collected Early in the SARS-CoV-2 Pandemic (First Follow-Up)

In order to evaluate the patients’ experiences of changes over the course of the SARS-CoV-2 pandemic, taking into account the patients’ health status, they were queried about the occurrence of MS relapses, MS progression, and comorbidities over the past three months. Additionally, the pwMS were requested to evaluate their degree of recent mental, physical, and social stress associated with the pandemic. The Trauma Screening Questionnaire (TSQ), a 10-item tool derived from the 17-item post-traumatic stress disorder (PTSD) symptom scale [51], was used to screen for PTSD [52,53]. It was adapted to assess the effects of the SARS-CoV-2 pandemic, with item wording tailored to the pandemic context, and the response scale modified into a 5-point scale (0 = “not at all”, 4 = “very strongly”). The TSQ includes five items on arousal and five on re-experiencing symptoms. To evaluate the impact of national SARS-CoV-2 measures and the risk of infection alongside MS, the 5-point scale was reclassified into a 2-point scale (0 = no (not at all, almost not, not sure), 1 = yes (strongly, very strongly)), and a sum score of 6 or higher was considered to be indicative of probable PTSD [53,54,55,56]. Furthermore, the patients were surveyed on the suitability of the policy measures implemented in response to the SARS-CoV-2 pandemic, with responses ranging from “highly inappropriate” to “highly appropriate”. Additionally, they were asked whether the pandemic had resulted in or exacerbated negative attitudes towards recommended standard vaccinations. With regard to the development of SARS-CoV-2 vaccines, pwMS were also queried regarding their willingness to be vaccinated against SARS-CoV-2 (unwilling, uncertain, willing).

#### 2.2.3. Data Collected After the Start of the SARS-CoV-2 Vaccination Campaign (Second Follow-Up)

In addition to the variables that were already surveyed in the first follow-up, the following variables were recorded in the second follow-up: the presence of SARS-CoV-2 infection during the pandemic (yes, no), the level of anxiety associated with the potential of infection with SARS-CoV-2 (no fear, light, medium, strong), the administration of vaccines other than those against SARS-CoV-2 in the last three months, and the number, date, and vaccine type of vaccinations received against SARS-CoV-2. At the time of the survey, the available vaccines in Germany included tozinameran, elasomeran, AZD1222, and Ad26.COV2.S [57].

### 2.3. Statistics

The full dataset comprised a total of 149 pwMS and 199 sociodemographic, clinical, and other variables. There were 154 variables from the baseline examination (from June 2019), 21 variables from the first follow-up survey (May 2020–July 2020), and 24 variables from the second follow-up survey (October 2021–January 2022). The patients were divided into 6 groups according to whether they intended to be vaccinated against SARS-CoV-2 at the first follow-up and whether they actually received such a vaccination before the second follow-up.

The descriptive and comparative analyses of the data were conducted in R version 4.1.2. For descriptive statistics, we calculated means and standard deviations (SD) for normally distributed continuous data, medians and ranges for non-normally distributed continuous data, and frequencies and percentages for categorical data. For inferential statistics, we utilized one-way analyses of variance (ANOVAs), Kruskal–Wallis tests, and chi-squared tests, respectively, to detect differences between the groups. All statistical tests were performed with cases with valid data, and, thus, cases with missing data were not considered. The significance level was set at α = 0.05 without correction for multiple testing, as all analyses were exploratory in nature.

Variables with significant differences between the patient groups at baseline or at the first follow-up were used to create a classification tree. This was done with the decision tree function from the parsnip R package. The model was fitted using the computational engine rpart with the default hyperparameters. More specifically, the minimum number of cases required to split a node was 20 and the penalty for tree complexity was 0.01.

## 3. Results

### 3.1. Patient Characteristics and Grouping of Patients According to Vaccination Willingness and Status

A total of 149 patients participated in all three surveys (i.e., at baseline and the two follow-ups). The average age of the patients was 48.4 ± 11.8 years (range: 20–74). The female–male ratio was 1.8. Approximately half of the patients were employed (49.7%), while 40.9% of the patients already received a disability pension. The majority of the patients were living in a partnership (76.5%). A relatively high proportion of the patients were current smokers (34.9%) or former smokers (29.5%). Relapsing MS (CIS or RRMS) was diagnosed in 69.1% of the patients, while 30.9% had progressive MS (SPMS or PPMS). The average degree of neurological disability on the EDSS was 3.5 points at a median disease duration of 10 years. The majority of the patients (73.8%) suffered from one or more secondary illnesses (e.g., cardiovascular or psychiatric diseases) in addition to MS. A DMT was used for the treatment of MS by 78.5% of the patients. The most frequently used DMTs were interferon beta (*n* = 20), natalizumab (*n* = 15), and fingolimod (*n* = 13). A total of 101 patients (67.8%) had already discontinued at least one DMT in the past, and only 12 patients (8.1%) were DMT-naive. Almost all patients (94.6%) were using drugs to mitigate MS-related symptoms or comorbidities. Overall, the patients were taking an average of 5.1 ± 3.0 drugs either as a prescription or as self-medication (Table 1).

With regard to vaccinations, only 26.8% of the pwMS reported at baseline that they had received general vaccination advice from a doctor in the past year, and 34.2% wished to receive such advice. According to the information in the vaccination cards, more than half of the patients had a complete vaccination status against pertussis (63.8%), diphtheria (67.1%), tetanus (67.8%), and poliomyelitis (85.2%). On the other hand, there were 81 patients (54.4%) who stated that they had never been vaccinated against the flu, often due to personal reasons (*n* = 66). Many, though not all, of the patients (*n* = 126, 84.6%) knew that there are governmental recommendations for vaccinations, and most of the patients (*n* = 112, 75.2%) were willing to undergo all officially recommended vaccinations prior to the SARS-CoV-2 pandemic. Reasons for the refusal of standard vaccinations at baseline included concerns about possible disease exacerbation by vaccines (*n* = 12), concerns about side effects of vaccinations (*n* = 9), and concerns that vaccinations would not be effective due to the medication the patient was taking (*n* = 5).

At the time of the first follow-up survey, some patients expressed some degree of uncertainty and frustration due to the public debate surrounding SARS-CoV-2: 10 patients (6.7%) stated that they had developed negative attitudes or more negative attitudes toward standard vaccinations, and 26 patients (17.4%) stated that the policy measures that had been implemented to control the SARS-CoV-2 pandemic were inappropriate. When asked specifically whether they would consider being vaccinated against SARS-CoV-2 once a vaccine was available, 33 patients (22.1%) responded that they were unwilling, while 23 patients (15.4%) were unsure and 93 patients (62.4%) were willing to receive such a vaccination.

**Figure 1:**
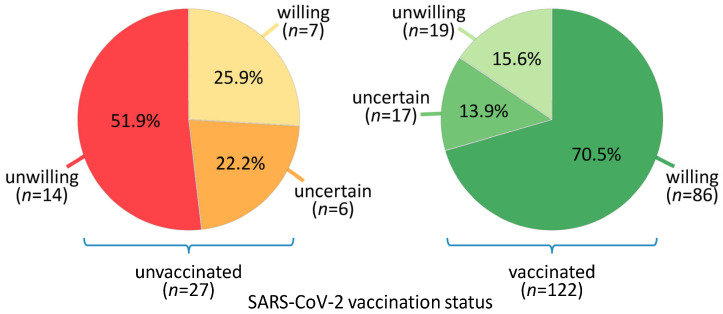
Vaccination willingness and vaccination status regarding SARS-CoV-2 in patients with MS (*N* = 149). The patients’ intention to get vaccinated against SARS-CoV-2 was surveyed between May and July 2020, before the first vaccines were authorized. About ~1.5 years later, we asked the patients whether they had been vaccinated against SARS-CoV-2 in the meantime. The Venn diagrams show the degree of discrepancy between the willingness to get vaccinated and the actual vaccination status. MS = multiple sclerosis, SARS-CoV-2 = severe acute respiratory syndrome type 2.

At the time of the last survey, a total of 122 patients (81.9%) had received a SARS-CoV-2 vaccination, and 27 patients (18.1%) were still unvaccinated. The most common reasons for vaccination were protection from a serious illness (87.7%), responsibility for the social environment (81.1%), and more freedom in daily life (52.5%). The most common reasons against vaccination were concerns about possible side effects (77.8%), the opinion that the vaccines had not yet been sufficiently tested (59.3%), and the fact that an infection is possible even after vaccination (55.6%) (multiple answers were possible). Interestingly, the SARS-CoV-2 vaccination willingness at the first follow-up was somewhat in discrepancy with the actual vaccination status at the second follow-up. Accordingly, the patients were divided into six groups based on their initial willingness to receive such a vaccination and their later vaccination status, as mRNA-based and vector-based SARS-CoV-2 vaccines had long been available by the time of the second follow-up: Among those unvaccinated patients were seven patients who had previously expressed their willingness to get vaccinated (unwilling: *n* = 14, uncertain: *n* = 6). On the other hand, there were also pwMS who were previously unwilling to be vaccinated (*n* = 19) or uncertain (*n* = 17) about being vaccinated but who nonetheless got vaccinated. The largest patient group (*n* = 86) comprised those who wanted to get vaccinated and also did get vaccinated against SARS-CoV-2 (Figure 1). Of note, only eight patients (5.4%) had experienced a SARS-CoV-2 infection at the second follow-up time point, and 56 patients (37.6%) stated that they were not afraid of such an infection. As in the previous survey, the public health measures that were put in place in response to the pandemic were viewed critically, with 37 of the patients (24.8%) considering them somewhat or totally inappropriate.

### 3.2. Factors Associated with Vaccination Willingness and/or Vaccination Status in Relation to SARS-CoV-2

**Figure 2:**
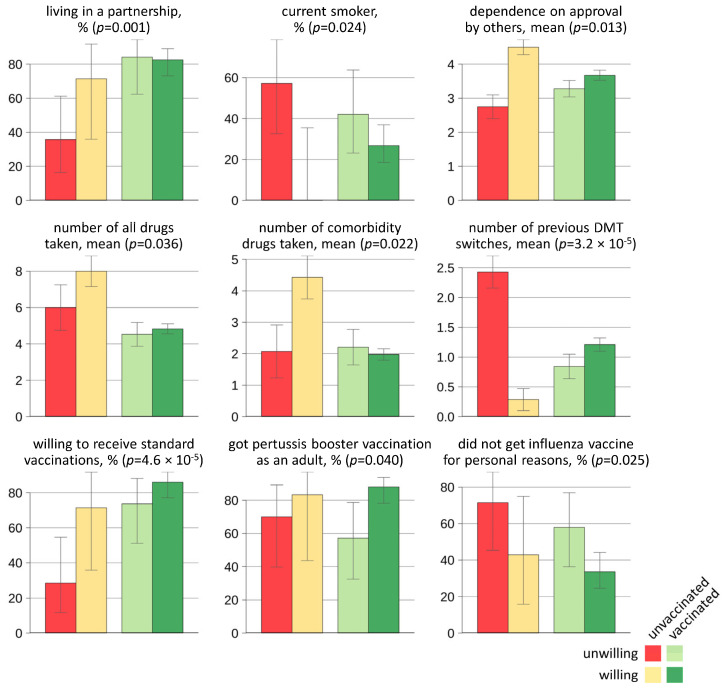
Differences in the baseline characteristics of MS patients (*N* = 149) in relation to SARS-CoV-2 vaccination willingness and status. Selected patient-specific factors, medication use patterns, and vaccination habits in which the patient groups differed significantly are presented. MS patients who were uncertain whether they would like to receive a SARS-CoV-2 vaccination before the first vaccines were authorized (i.e., at first follow-up) are not included in this figure for better clarity (see Supplementary Table S1 for the full results). Shown are proportions with 95% Wilson score confidence intervals for categorical variables and means ± standard errors for numerical variables. Only valid (i.e., non-missing) data were taken into account for the statistical analyses. DMT = disease-modifying therapy, MS = multiple sclerosis, SARS-CoV-2 = severe acute respiratory syndrome type.

In total, 16 variables from the baseline data demonstrated significant associations with the grouping of the patients. The full results of our exploratory univariable analyses can be found in Supplementary Table S1. Selected data are visualized in Figure 2 and summarized in the following: Among the demographic variables, we found that pwMS who did not intend to be vaccinated against SARS-CoV-2 and who also did not receive such a vaccination were significantly less likely to live in a partnership (35.7%). A larger proportion of the patients who did not want to be vaccinated were current smokers compared to those who wanted to be vaccinated (among those who were unvaccinated: 57.1% vs. 0.0%, among those who were vaccinated: 42.1% vs. 26.7%). Patients who did not want to be vaccinated also had lower average scores on the TCI-R subscale dependence on the consent of others. The medication profile of the patients was identified as another potentially important factor in this context: Patients who were unvaccinated despite previously expressing their willingness to get vaccinated took on average a particularly high number of medications (8.0 ± 2.2), especially medications for comorbidities (4.4 ± 1.8). Moreover, the number of previously discontinued DMTs for MS was significantly higher among those who were unvaccinated and unwilling to get vaccinated (2.4 ± 1.0) than in the other groups. Among these, only a few stated in the baseline survey that they were willing to receive the recommended standard vaccinations (28.6%), with two patients declaring that they believe that vaccinations are generally ineffective due to their medication. Furthermore, patients who were unwilling to get vaccinated against SARS-CoV-2 had less often received a pertussis booster vaccination as an adult and more often did not receive a vaccination against influenza for personal reasons. It should also be noted that several variables showed no significant association with SARS-CoV-2 vaccination willingness and status, including age, sex, employment status, disease duration, course of disease, NEO-FFI personality traits, and measures of anxiety and depression.

**Figure 3:**
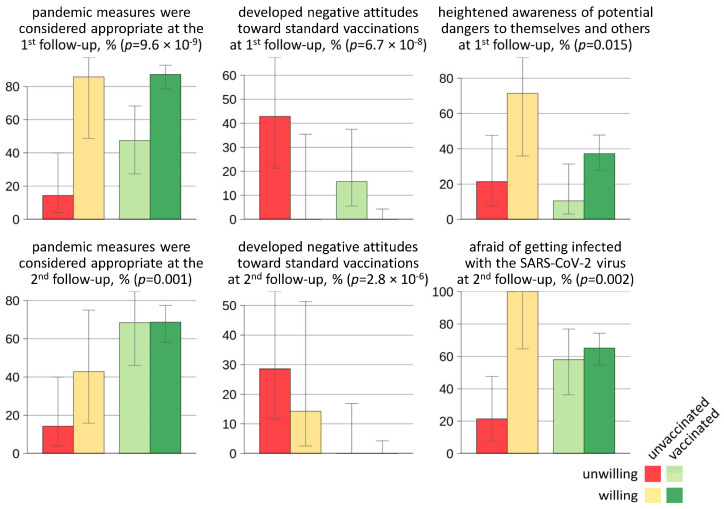
Differences between the groups of pwMS identified in the two follow-up surveys. There were significant differences in opinions on the measures that were implemented during the coronavirus 2019 pandemic, attitudes toward recommended vaccinations, and perceptions of risks. Patients with MS who were uncertain whether they would like to receive a SARS-CoV-2 vaccination before the first vaccines were authorized (i.e., at first follow-up) are not included in this figure for better clarity (see Supplementary Table S1 for the full results). Shown are proportions with 95% Wilson score confidence intervals. MS = multiple sclerosis, SARS-CoV-2 = severe acute respiratory syndrome type 2.

In the follow-up surveys, there were eight variables with significant differences between the six patient groups (Supplementary Table S1). On the one hand, those who were unvaccinated and unwilling to get vaccinated rarely stated that they consider the measures for managing the pandemic to be appropriate (14.3% at both time points). They reported significantly more often that they had developed a negative attitude or had a more negative attitude toward standard vaccines because of the SARS-CoV-2 pandemic (first follow-up: 42.9%, second follow-up: 28.6%). Patients who were willing to get vaccinated against SARS-CoV-2 more often stated that they have a heightened awareness of potential dangers to themselves and others and that they are afraid of getting infected with the coronavirus (Figure 3). In addition, patients who were uncertain about being vaccinated more frequently reported bodily reactions when reminded of the pandemic event (26.1%), and some of them (17.4%) reported another illness in the three months prior to the first follow-up.

### 3.3. Classification of MS Patients According to SARS-CoV-2 Vaccination Willingness and Status

**Figure 4:**
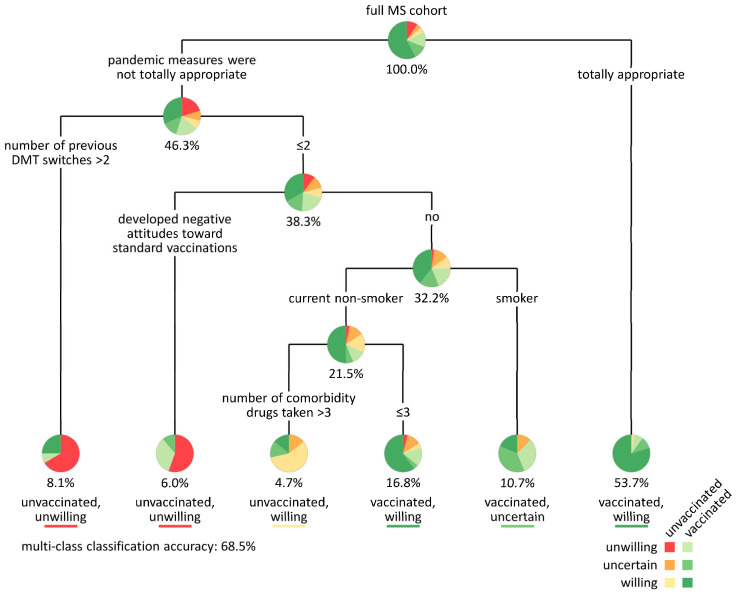
Classification tree for categorizing patients with MS (*N* = 149) according to vaccination willingness and status. For the decision tree modeling, we considered all variables in which the patient groups differed significantly at baseline or at first follow-up. The resulting decision tree achieved an accuracy of 68.5% with a depth of 5. The pie charts show the proportion of subgroups for each node. Class assignment at leaf nodes is based on the majority vote. Eighty patients found the public health measures to control the spread of SARS-CoV-2 totally appropriate. Most of these patients (80.0%) were willing to get vaccinated at the first follow-up and received a vaccination before the second follow-up (right branch). Patients who were unwilling to receive such a vaccination had typically discontinued at least three different DMTs for MS in the past and had developed or enhanced negative attitudes toward generally recommended vaccinations during the pandemic. DMT = disease-modifying therapy, MS = multiple sclerosis, SARS-CoV-2 = severe acute respiratory syndrome type.

The factors that showed significant differences between the six patient groups at baseline (*n* = 16) or at the first follow-up (*n* = 5) were used to construct a decision tree (Figure 4). As a result, the patients were first split according to how they rated the policy measures that had been implemented during the COVID-19 pandemic. Eighty patients (53.7%) considered these measures totally appropriate, and most of them (*n* = 64, 80.0%) were vaccinated and willing to be vaccinated, while only two of them (2.5%) were not vaccinated. The remaining patients were further divided depending on how often they had switched their DMT in the past. Eight of the 12 patients (66.7%) who did not consider the measures totally appropriate and who had more than two DMT switches were unvaccinated and unwilling to get vaccinated. Other independent variables that were included in the model were the development or enhancement of negative attitudes toward routine vaccinations, current smoking status, and the number of drugs taken to treat comorbidities. Overall, the multivariable classification using the decision tree achieved an accuracy of 68.5%.

## 4. Discussion

The objective of this study was two-fold: first, the objective was to compare the SARS-CoV-2 vaccination status in pwMS with their reported willingness to become vaccinated, and, second, it was to determine factors that are associated with the decision of whether to get vaccinated. We found that 75.2% of the pwMS accepted the standard vaccinations recommended by the German Standing Committee on Vaccination (STIKO) [58]. However, there was a discrepancy between the proportion of patients who had declared their intention to be vaccinated against SARS-CoV-2 (62.4% in the period from May to July 2020) and the proportion who were later actually vaccinated (81.9% in the period from October 2021 to January 2022). The observed SARS-CoV-2 vaccination rate of 81.9% is consistent with findings from other studies, such as the finding of 82.9% in an Australian MS cohort [59] and that of 90.3% in a Spanish study [60]. In Iran, the vaccine uptake varied, with 90.7% of patients with RRMS and 79.3% of those with SPMS being vaccinated [21]. While the initial willingness to be vaccinated was high in some groups, the actual uptake was significantly lower in several cases.

We identified several factors that distinguished patients who were willing or unwilling to receive a SARS-CoV-2 vaccination and those who were later vaccinated or not vaccinated. These insights can help healthcare providers to better understand and address vaccine hesitancy. This may ultimately improve vaccination rates and may improve outcomes, particularly in patients receiving B-cell depleters, although this needs to be confirmed in future studies.

Firstly, we will examine the factors that led patients to adopt a positive vaccination behavior against SARS-CoV-2. In the present study, pwMS with fewer comorbidities, fewer medications, and fewer DMT switches were found to be more likely to be vaccinated. To the best of the present author´s knowledge, it is not apparent that any other study has arrived at conclusions similar to those outlined here. It is hypothesized that these patients may experience a reduced risk of complications from vaccination or a heightened sense of safety overall. PwMS with the aforementioned characteristics may be healthier overall, thus increasing their confidence in a positive effect of vaccination. These results are in contrast to a study by Inojosa et al., which found that the proportion of pwMS who had received at least two doses of a SARS-CoV-2 vaccine was significantly higher among older pwMS, patients with comorbidities patients, and those with more severe disability [61]. In a study by Serrazina et al. [62], pwMS who were older and had more comorbidities were also more willing to become vaccinated. This discrepancy underscores the challenge of accurately delineating patients who decline vaccination, thereby highlighting the intricacies inherent in the decision-making process.

Previous vaccination behavior has been demonstrated to offer insights into the propensity of an individual to receive a vaccination against SARS-CoV-2. In our study, existing booster vaccinations against pertussis and influenza were identified as significant positive predictors (>80% of those vaccinated vs. <65% of those unvaccinated). One potential explanation for this phenomenon is that individuals who have received all recommended vaccine doses, such as those for influenza, tend to hold a favorable attitude towards vaccination in general [31]. It can be posited that such individuals may be more amenable to protective measures and place greater trust in the recommendations of medical professionals. In the period preceding the pandemic, pwMS may have recognized the advantages of vaccination for themselves and consequently exhibited a greater propensity to receive the SARS-CoV-2 vaccination. A multitude of studies have demonstrated that an individual’s vaccination behavior prior to the pandemic serves as a predictor of their inclination to receive a SARS-CoV-2 vaccine. Previous positive vaccination behavior and frequent utilization of healthcare services have been identified as factors that contribute to a higher propensity for vaccination [17,18,19,28,29,30].

PwMS in partnership relationships also demonstrated a greater propensity to receive the vaccination against SARS-Cov-2. The underlying factors contributing to this phenomenon may include the provision of support and motivation from partners. Partnerships have been shown to enhance feelings of security and cohesion, which in turn makes individuals more likely to adhere to health recommendations, such as those regarding vaccinations. Furthermore, the dissemination of information regarding the advantages of vaccination by one’s partner has been demonstrated to enhance the confidence of the patient that they are making an informed decision regarding vaccination. It is also conceivable that this observation is related to the sources of information obtained from pwMS, which might have a positive influence on their willingness to become vaccinated. Patients who obtained information from news outlets, charitable organizations, and family members were at least two times more likely to be vaccinated than those who obtained information from other sources [17]. Proietti et al. [28] also found a positive correlation between the willingness to become vaccinated and a trusting relationship with the treating physician and the healthcare system in general. The most frequently cited reasons for SARS-CoV-2 vaccination in our MS cohort were protection against serious illness (87.7%), responsibility for the social environment (81.1%), and more freedom in daily life (52.5%). Similar reasons for positive vaccination attitudes were reported by Uhr and Mateen [18].

In the following, we aim to elucidate the factors that, according to our findings, have contributed to a negative attitude towards vaccination. The number of overall medications and comorbidity-related medications was found to be particularly high in the SARS-CoV-unvaccinated pwMS. This is consistent with the findings of another study, where individuals with higher levels of functional impairment were observed to exhibit a greater reluctance to receive a SARS-CoV-2 vaccination [62]. Moreover, the number of previous DMT switches was found to be significantly higher in the unvaccinated pwMS who were also unwilling to become vaccinated. There is no direct evidence from the literature that links the willingness to receive a SARS-CoV-2 vaccine with the number of DMT switches in pwMS, as only the percentage of respondents who are on DMTs is provided [18,19,21,29,62,63,64]. The underlying reasons for these observations may be attributable to the concerns of these pwMS regarding potential adverse effects or interactions of the vaccination with their current therapeutic regimens, given their complex medical histories and the multiple medications they are prescribed. The potential repercussions of the vaccination, including the possibility of exacerbating their MS or the vaccination interfering with their current treatment regimen, may be a source of concern for some individuals. Furthermore, individuals with prior experience of adverse effects or complications arising from medical treatments may exhibit a degree of skepticism or uncertainty with regard to new vaccinations. This is consistent with our findings that the most common reasons for being against vaccination were concerns about possible side effects (77.8%). In addition, the complexity of an individual’s regimen, characterized by a multitude of medications and treatment modifications, has the potential to engender feelings of uncertainty or trepidation among that individual. This may, in turn, contribute to an unfavorable stance towards vaccination. The belief that the vaccines have not been sufficiently tested (59.3%) and that an infection is possible even after vaccination (55.6%) were commonly cited reasons for refusing vaccination. Nabavi et al. reported no correlation between vaccination behavior and the number of comorbidities or previous DMTs [21]. In contrast, Huang et al. found that pwMS who are using DMTs have a 1.7 times higher propensity to be vaccinated than those who are not using DMTs [17]. These observations, which appear to be somewhat contradictory, underscore the necessity for additional research that encompasses a more substantial cohort of patients. The findings of the study by Day et al. [20] indicate that disease-related considerations were of paramount importance to medically vulnerable populations and influenced their decision to become vaccinated against SARS-CoV-2. The reasons cited for vaccination against SARS-CoV-2 are primarily related to medical concerns [17,18,19,20,21,28,29,30,32,62,63,65,66,67,68,69,70,71,72,73]. A considerable proportion (greater than 50%) of the subjects cited safety concerns as a rationale for declining the vaccination. This reservation was also observed in other studies [18,29,68,74,75]. It is therefore imperative that patients are adequately informed about the safety of vaccines.

Lifestyle factors have been demonstrated to be a contributing element in the decision-making process regarding vaccination [76]. In the present study, the vaccination opponents were more likely to live alone. It is hypothesized that single pwMS are more likely to be unvaccinated due to a lack of social support, which may leed them to choose not to vaccinate or to not seek advice on questions and uncertainties. Furthermore, it is suggested that these patient groups may feel less well informed due to having a smaller social network, or may have more doubts about the safety and effectiveness of vaccination. Furthermore, the subject may encounter a paucity of assistance in their day-to-day existence, thereby hindering the discussion and resolution of potential fears or concerns. Consequently, it is imperative to provide them with targeted information and support to foster their willingness to be vaccinated. A study of the impact of pre-pandemic social isolation on self-rated health during the pandemic reveals that social isolation had a detrimental effect on health outcomes during this period in Japan. This finding suggests the need for policy measures aimed at preventing social isolation to enhance public health resilience [77]. Furthermore, our results indicated that pwMS who smoke were less inclined to receive such vaccination. The latter is in line with the findings by Mhereeg et al. [78] who studied pregnant women in Wales who were smokers. Jackson et al. conducted a survey of the general population and found that current smokers generally have a more negative attitude towards vaccination against SARS-CoV-2 [79]. The study revealed that attitudes varied by smoking history, with a more negative attitude being reported by 10.6% of current smokers, 8.8% of former smokers, and 5.9% of never smokers. Among those who were currently smoking, the percentage was 17.7%. However, we could not confirm previously reported disparities that were observed in the vaccination rates of individuals of different demographics.

We also found that lower TCI-R scores on the question of dependence on the opinion of others were associated with unwillingness to get vaccinated and remaining unvaccinated against SARS-CoV-2. This phenomenon could potentially signify a diminution in the influence exerted by the prevailing thought and conduct of the majority of the general population. Our analysis revealed significant differences in the perception of pandemic measures, attitudes toward routine vaccinations, and the risk perception of SARS-CoV-2 between vaccinated and unvaccinated pwMS. Notably, a considerable proportion of unvaccinated pwMS (28.6% by the second follow-up) developed negative attitudes towards standard vaccinations, whereas this was not apparent among vaccinated individuals. Only 14.3% of the pwMS who were both unwilling and unvaccinated considered governmental measures appropriate, with this attitude emerging as a key predictor of vaccination status. Fear of infection was more prevalent among vaccinated pwMS (approximately 60%) than among unwilling as well as unvaccinated individuals (21.4%), which aligns with previous studies indicating that subjective norms, fear of the virus, and vaccine confidence strongly influence vaccination willingness. A lack of willingness to vaccinate was related to a reduced level of concern regarding infection by the novel pathogen SARS-CoV-2, and a diminished perception of the impact of the resulting disease on the lives of those who are infected was found in a study by Serrazina et al. [62]. A study by Gilan et al. indicated that individuals with elevated concerns regarding the vaccination were more likely to remain unvaccinated [80]. According to Oniszczenko et al., fear of infection and fear of the vaccine for SARS-CoV-2 are positively correlated [81], whereas Mertens et al. determined that fear of SARS-CoV-2 infection was a significant predictor of willingness to become vaccinated against SARS-CoV-2 [82]. These findings highlight the need for targeted communication strategies to effectively address vaccine hesitancy in this population.

It is worth noting that our study is not without limitations. This study was conducted in two hospitals in central and northern Germany, which represents a geographical limitation. Specifically, we only collected data from patients in clinical MS centers; the private practice sector was excluded in our analysis. This study focuses on individuals with MS in eastern Germany, a demographic that is often underrepresented in research. In Thuringia, the rate of vaccination against SARS-CoV-2 in the general population in March 2024 was 76.4%, which was lower than the 84.5% recorded in West Germany (for example, Bremen). This discrepancy may be attributed to deep-rooted mistrust in state institutions, influenced by the historical context of the GDR, the heightened influence of anti-vaccination and populist sentiments, and socio-economic factors such as elevated unemployment and future job insecurity, which can collectively contribute an environment that is conducive to vaccine skepticism [83]. While the sample size was sufficient to ensure adequate statistical power, a larger sample would have been desirable, as it would enhance the validity of the results. The findings of our study are limited to the SARS-CoV-2 vaccination situation in Germany. It should be noted that the situation may differ in other countries and for other vaccinations. To gain a more nuanced understanding of vaccine hesitancy, it is crucial to identify strategies that promote confidence in vaccination by increasing trust in the healthcare system and reducing misconceptions. Overcoming vaccine hesitancy is one of the most significant challenges that public health is currently facing. The reasons for the disparate choices observed in pwMS are complex and multifaceted, as evidenced by the findings of this study. The results of our study could contribute to a more comprehensive understanding of vaccine hesitancy during the SARS-CoV-2 pandemic and thus support the refinement of public health strategies to improve preparedness for future pandemics. The resurgence of higher SARS-CoV-2 infection rates underscores the necessity for more comprehensive counseling and clarification of potential vaccine hesitancy [84]. It is essential to acknowledge concerns and provide personalized information to cultivate confidence and encourage acceptance of vaccination. This incongruity highlights the challenge of more precisely delineating patients who decline vaccination and underscores the intricacy of the decision-making process. Consequently, the execution of further studies with a more substantial sample size is essential to ensuring a comprehensive understanding of the phenomenon under investigation.

## 5. Conclusions

The merits of this study lie in the confidentiality that was ensured during the collection of comprehensive clinical and demographic data as well as the attitudes of the respondents toward vaccinations. The responses regarding the willingness to receive a SARS-CoV-2 vaccine were collected prior to the widespread distribution of such vaccines. It is of the utmost importance to gain a better understanding of the underlying causes and key factors that contribute to vaccine rejection. Informing patients about necessary vaccinations and their associated health benefits is the responsibility of doctors. The majority of the pwMS analyzed in this study had received a SARS-CoV-2 vaccination. The pwMS who declined vaccination against SARS-CoV-2 were more often current smokers, and had, on average, lower scores on the TCI-R subscale dependence on the consent of others. Furthermore, the patients demonstrated a diminished inclination to be vaccinated against SARS-CoV-2 compared to their willingness to become vaccinated against other diseases. It is the responsibility of neurologists to inform vaccine-hesitant pwMS about the long-term consequences of SARS-CoV-2 and to emphasize the fact that previous pandemics have been eradicated by common vaccinations. Furthermore, they may wish to cite studies indicating that there is no significant difference between the rates of adverse effects associated with the administration of the vaccines to individuals with neuroinflammatory diseases and those associated with their administration to control groups [85]. Furthermore, additional pertinent information regarding vaccine safety, potential self-limiting side effects, and the vaccine’s ability to prevent a severe illness is crucial to allowing informed decision-making and increasing vaccination acceptance. The patients’ concerns should be taken seriously so that they can choose to become vaccinated based on scientific evidence and information from a healthcare provider that they perceive as caring and attentive.

## Figures and Tables

**Table 1:** Characteristics of the patient cohort (*N* = 149).

Characteristic	Statistics
**Age (years), mean ± SD**	48.4 ± 11.8
**Sex, *n* (%)**	
Women	96 (64.4)
Men	53 (35.6)
**Employment status, *n* (%)**	
Full-time work	42 (28.2)
Part-time work	32 (21.5)
Unemployed	5 (3.4)
Old-age pension	9 (6.0)
Disability pension	61 (40.9)
**Partnership, *n* (%)**	
No	35 (23.5)
Yes	114 (76.5)
**Current smoker, *n* (%)**	
No	97 (65.1)
Yes	52 (34.9)
**Disease course, *n* (%)**	
CIS	8 (5.4)
RRMS	95 (63.8)
SPMS	36 (24.2)
PPMS	10 (6.7)
**Disease duration (years), median (range)**	10 (0–37)
**EDSS score, median (range)**	3.5 (0.0–8.5)
**Patient care, *n* (%)**	
Outpatient	122 (81.9)
Inpatient	27 (18.1)
**Comorbidities, *n* (%)**	
0	39 (26.2)
1	28 (18.8)
≥2	82 (55.0)
**Number of drugs taken, *n* (%)**	
0	1 (0.7)
1–4	71 (47.7)
5–9	65 (43.6)
≥10	12 (8.1)
**DMT use, *n* (%)**	
No	32 (21.5)
Yes	117 (78.5)

CIS = clinically isolated syndrome, DMT = disease-modifying therapy, EDSS = Expanded Disability Status Scale, MS = multiple sclerosis, PPMS = primary progressive multiple sclerosis, RRMS = relapsing-remitting multiple sclerosis, SD = standard deviation, SPMS = secondary progressive multiple sclerosis.

## Data Availability

The datasets generated and analyzed in the current study are available from the corresponding author on reasonable request.

## Supplementary Materials

The following supporting information can be downloaded at: https://www.mdpi.com/article/10.3390/jcm14113689/s1, Table S1: Factors associated with vaccination willingness and/or vaccination status in relation to SARS-CoV-2.

## Abbreviations

The following abbreviations are used in this manuscript:

pwMS	People with Multiple Sclerosis
PTSD	Post-traumatic stress disorder
CIS	Clinically isolated syndrome
DMT	Disease-modifying therapy
EDSS	Expanded Disability Status Scale
MS	Multiple Sclerosis
PPMS	Primary progressive Multiple Sclerosis
RRMS	Relapsing-remitting Multiple Sclerosis
SD	Standard deviation
SPMS	Secondary progressive Multiple Sclerosis
SARS-CoV-2	Severe Acute Respiratory Syndrome type 2
